# An Online Nanoinformatics Platform Empowering Computational Modeling of Nanomaterials by Nanostructure Annotations and Machine Learning Toolkits

**DOI:** 10.1021/acs.nanolett.4c02568

**Published:** 2024-08-09

**Authors:** Tong Wang, Daniel P. Russo, Philip Demokritou, Xuelian Jia, Heng Huang, Xinyu Yang, Hao Zhu

**Affiliations:** † Tulane Center for Biomedical Informatics and Genomics, Tulane University, New Orleans, Louisiana 70112, United States; ‡ Division of Biomedical Informatics and Genomics, Deming Department of Medicine, Tulane University, New Orleans, Louisiana 70112, United States; § Department of Chemistry and Biochemistry, Rowan University, Glassboro, New Jersey 08028, United States; ∥ Center for Nanotechnology and Nanotoxicology, Department of Environmental Health, T.H. Chan School of Public Health, Harvard University, 655 Huntington Ave, Boston, Massachusetts 02115, United States; ⊥ Nanoscience and Advanced Materials Center, Environmental Occupational Health Sciences Institute, School of Public Health, Rutgers University, Piscataway, New Jersey 08854, United States; # Department of Computer Science, University of Maryland College Park, College Park, Maryland 20742, United States

**Keywords:** Nanoinformatics, Public databases, Nanostructure annotation, Machine learning, Predictive modeling

## Abstract

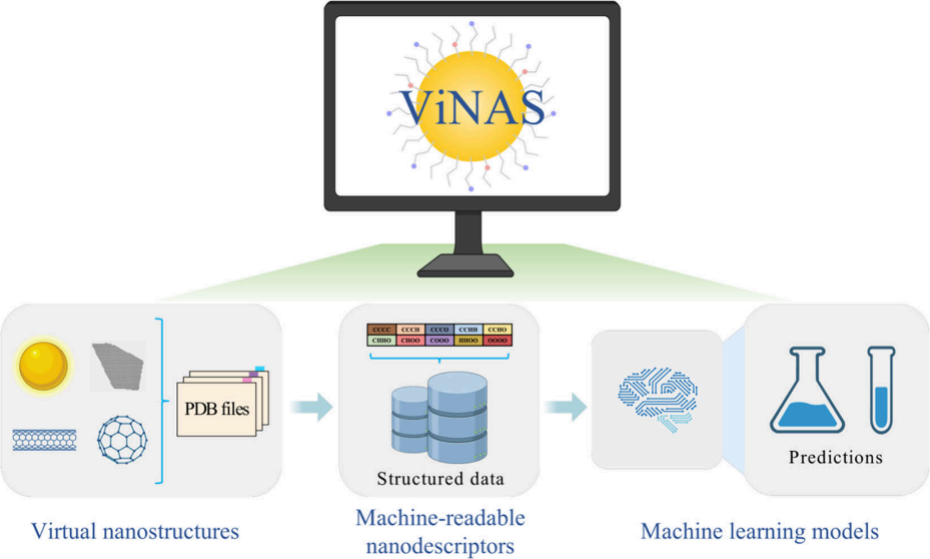

Modern nanotechnology has generated numerous datasets from *in vitro* and *in vivo* studies on nanomaterials, with some available on nanoinformatics portals. However, these existing databases lack the digital data and tools suitable for machine learning studies. Here, we report a nanoinformatics platform that accurately annotates nanostructures into machine-readable data files and provides modeling toolkits. This platform, accessible to the public at https://vinas-toolbox.com/, has annotated nanostructures of 14 material types. The associated nanodescriptor data and assay test results are appropriate for modeling purposes. The modeling toolkits enable data standardization, data visualization, and machine learning model development to predict properties and bioactivities of new nanomaterials. Moreover, a library of virtual nanostructures with their predicted properties and bioactivities is available, directing the synthesis of new nanomaterials. This platform provides a data-driven computational modeling platform for the nanoscience community, significantly aiding in the development of safe and effective nanomaterials.

Modern nanotechnology has been skyrocketing in research and commercial applications over the past decades.^1−4^ The global nanotechnology market is expected to exceed $290 billion in 2028.^5,6^ Nanomaterials (NMs) have been utilized in over 5,300 commercial products across almost all fields, such as agriculture,^7−9^ food processing,^10−12^ clean energy,^13^ and clinical medicine.^14,15^ In clinic, nanomedicine offers various advantages over conventional drugs, such as targeted delivery, enhanced solubility and absorption, controlled drug release, and protection from rapid clearance.^16^ Notably, nanotechnology has played a crucial role in two mRNA-based vaccines to combat the global COVID-19 pandemic.^17^ Nanoparticles used in these vaccines enhance stability, protect mRNA from degradation, and facilitate precise delivery to the target site.^17,18^ However, subtle structural modifications can lead to significant changes in NM’s properties and bioactivities, indicating a major challenge, making the design of new NMs and associated experimental evaluation costly and time-consuming. Moreover, the increasing usage of NMs raises great concerns regarding their impacts on human health and environments, which are designated as having “nanotoxicities” on ecosystems, humans, and other organisms.^19−22^ There is an urgent need for a new strategy for assessing both existing and emerging NMs’ properties/bioactivities/toxicities. Computational modeling, especially that based on machine learning (ML) approaches, is a promising strategy for rapid evaluation of new NMs by revealing quantitative relationships between the structural features of known NMs and their biological activities.^23−28^ Various *in vitro* and *in vivo* studies evaluating NMs’ properties/bioactivities/toxicities have generated numerous data available for computational modeling of NMs.^29,30^ According to the EU-US Nanoinformatics Roadmap 2030 guidance for ML model development, NM modeling relies heavily on high-quality NM data, nanodescriptors encoding NM properties, and user-friendly ML tools.^31^ This guidance emphasizes that the absence of public databases for computational modeling and model sharing is a significant challenge for nanoinformatics modeling.

PubChem and Protein Data Bank (PDB) are two large databases that have been widely used in the scientific community.^32−34^ PubChem maintains data regarding the structures, physicochemical properties, and bioactivities of small molecules. PDB stores three-dimensional structural data of biological macromolecules, primarily proteins and nucleic acids. These popular databases serve various research fields such as toxicology, structural biology, and computational biology.^35−40^ Particularly, ML-based computational modeling for small molecules, proteins, and nucleic acids has had significant advances with the curated data provided by these databases.^34,41^ For example, DeepMind’s AlphaFold2 accurately predicted protein 3D structures from amino acid sequences by training on 170,000 protein structures from PDB database using neural networks.^37^

Several NM databases have been designed to meet different needs within the nanotechnology community. For example, Cancer Nanotechnology Laboratory (caNanoLab) was created by the National Cancer Institute to provide the characterization and bioactivity data of NMs in the biomedical nanotechnology field.^42^ eNanoMapper was developed by the European Commission’s Seventh Framework Programme to support the safety assessments of engineered NMs.^43^ The NanoInformatics Knowledge Commons (NIKC) is a cyberinfrastructure that includes a data repository and associated analytical tools developed to visualize and interrogate integrated datasets.^44^ The database employs the “Knowledge and Instance Mapping” technique to guide the curation and integration of experimental metadata essential for characterizing each step in the transformation of NMs, with a focus on the relationships between NMs and their environments.^45^ This approach also aids in harmonizing data across various platforms such as NanoInformaTIX and NaKnowBase. NanoInformaTIX is a risk assessment modeling platform for engineered nanomaterials (ENMs), offering online data analysis and predictive tools.^44^ The EPA NaKnowBase is an SQL database that contains materials obtained from published research relevant to the potential environmental and biological impacts of ENMs.^46^ Current NM databases have made promising progress in facilitating data sharing (Table S1). However, many databases were rarely used in ML studies due to their limitations in computational modeling. For example, some databases are not fully accessible to the public. Information related to NM entities such as properties, composition, and bioactivities is often extracted directly from experimental studies, lacking the essential nanostructure annotation required for converting the structure data into machine-readable formats. To resolve these issues, in one of our recent studies, we developed Public Virtual Nanomaterial Simulation (PubVINAS), an NM database containing nanostructure and endpoint data suitable for modeling purposes^47^ (Table S1). However, most existing nanoinformatics portals, including PubVINAS, lack user-friendly tools for researchers to perform data analysis and modeling. Furthermore, all of these databases share data collected from existing NMs but do not provide new NMs with new nanostructures that can guide rational NM design.

To answer all the above challenges, we developed the Virtual Nanostructure Simulation Professional (ViNAS-Pro) platform that contains (1) machine readable and downloadable data of nanostructures, nanodescriptors, and assay testing results for a variety of NMs, (2) a nanostructure data analysis toolkit, (3) an ML modeling toolkit, and (4) a large virtual library of new NMs with predicted properties and activities (Figure S1, Table S1). ViNAS-Pro maintains two databases covering 14 types of NMs, providing their structural details as well as experimentally assessed property and biological activity data. ViNAS-Pro toolkits allow for easy data preprocessing, visualization, and predictive modeling. ViNAS-Pro virtual library provides property and/or bioactivity predictions for virtual NMs. ViNAS-Pro portal is a comprehensive nanoinformatics platform that provides high-quality data, user-friendly modeling tools, and property/activity/toxicity predictions and can directly support rational design of new NMs.

The ViNAS-Pro database provides high-quality, curated data for nano community research. The database provides rigorously curated assay data from the Nanotechnology Health Implication Research (NHIR) consortium, research papers, and other public sources. Data were collected and added to ViNAS-Pro database only when curation criteria were satisfied, such as (1) providing material information (e.g., core atoms) and size information; (2) annotating surface ligand structures and converting them into SMILES; and (3) providing nano-bioactivity or physicochemical property data with detailed experimental information. The nanostructures on ViNAS-Pro were annotated in standardized PDB formats that include atomic coordinates, chemical bonds, and other relevant structure data, which are easily accessible and can be used for various computational tasks, such as molecular dynamic simulations. Visualization of representative NMs’ structures in the database is shown in Figure 1. Based on PDB files consisting of accurately annotated nanostructures, NMs can be quantified as nanodescriptors for ML modeling of NMs’ properties/bioactivities/toxicities. The development of nanodescriptors focuses on the surface chemistry of NMs, which is crucial for NMs’ properties/bioactivities/toxicities.

**Figure 1:**
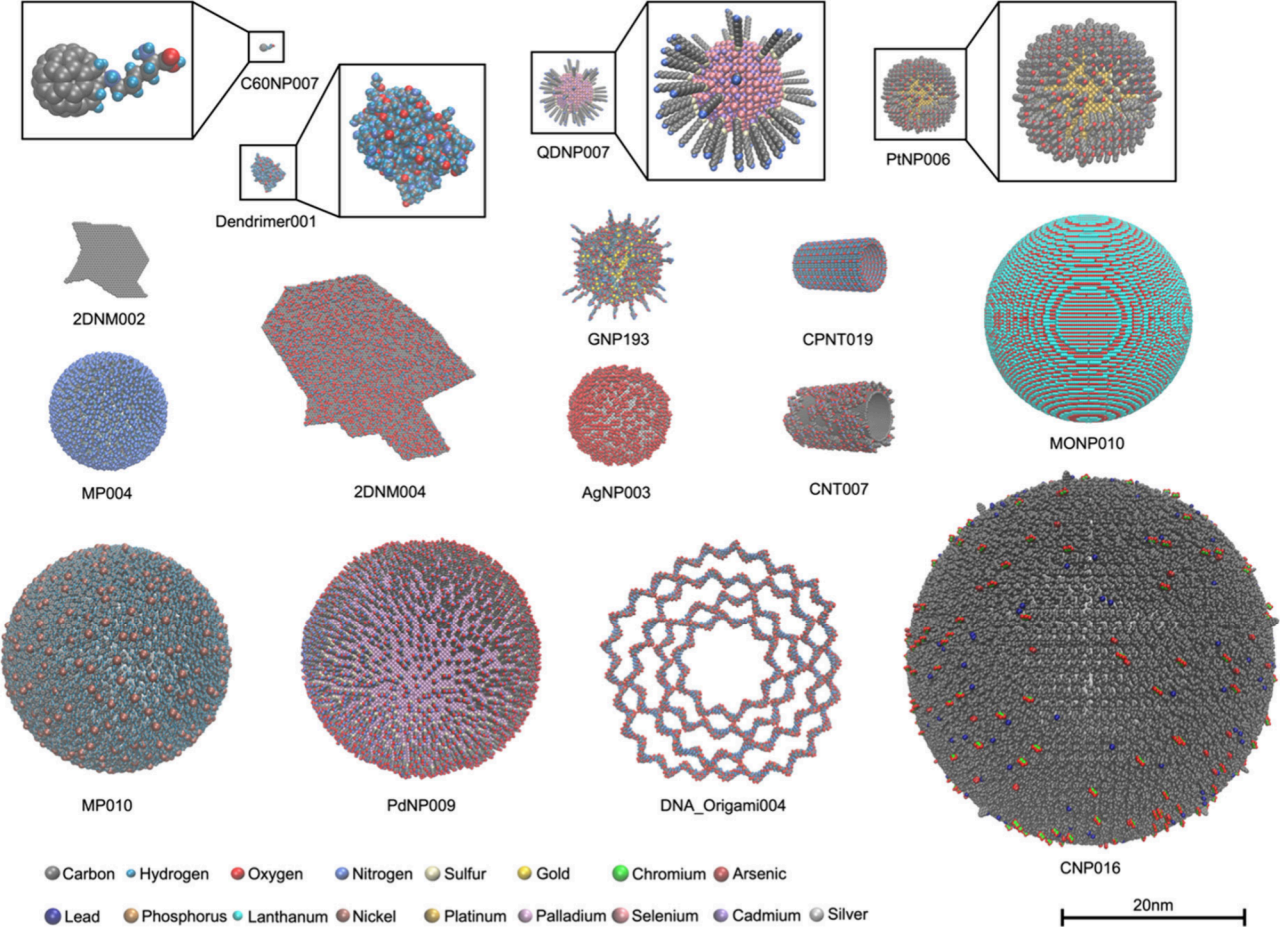
Visualization of representative NMs in the database. NMs on ViNAS-Pro belong to 14 material types, including gold nanoparticles, silver nanoparticles, platinum nanoparticles, palladium nanoparticles, buckminsterfullerenes, carbon nanoparticles, carbon nanotubes, dendrimers, DNA origami nanoparticles, metal oxide nanoparticles, quantum dots, cyclic peptide nanotubes, two-dimensional nanomaterials, and microplastics. The nanostructures were rendered using the VDW drawing method in visual molecular dynamics (VMD). Text represents identifiers that can be used to search for the NMs on ViNAS-Pro.

The assay database contains data from 27 assays conducted to test various NMs. An overview of the assays on ViNAS-Pro is shown in Table S2. A total of 21 assays predominantly assess human health impacts, while assays including PFOS adsorption and immobilization rate in *Daphnia magna* mainly focus on environmental concerns. Additionally, Zeta Potential assays primarily evaluate NM properties, which can influence both human health and environments. For example, a record for assay 18 (NanoAID-18) is shown in Figure 2A. Its assay record page provides a figure displaying the activity distributions of NMs tested against NanoAID-18 and an interactive table containing the results of NMs associated with NanoAID-18. The assay data and the associated NMs’ nanodescriptor data are both available for download as Microsoft Excel Open XML Spreadsheet (XLSX) files on the corresponding assay record page. Moreover, the endpoint definition, experimental protocol, and related literature are displayed on the assay record page, providing useful information for the assessment of new NMs.

**Figure 2:**
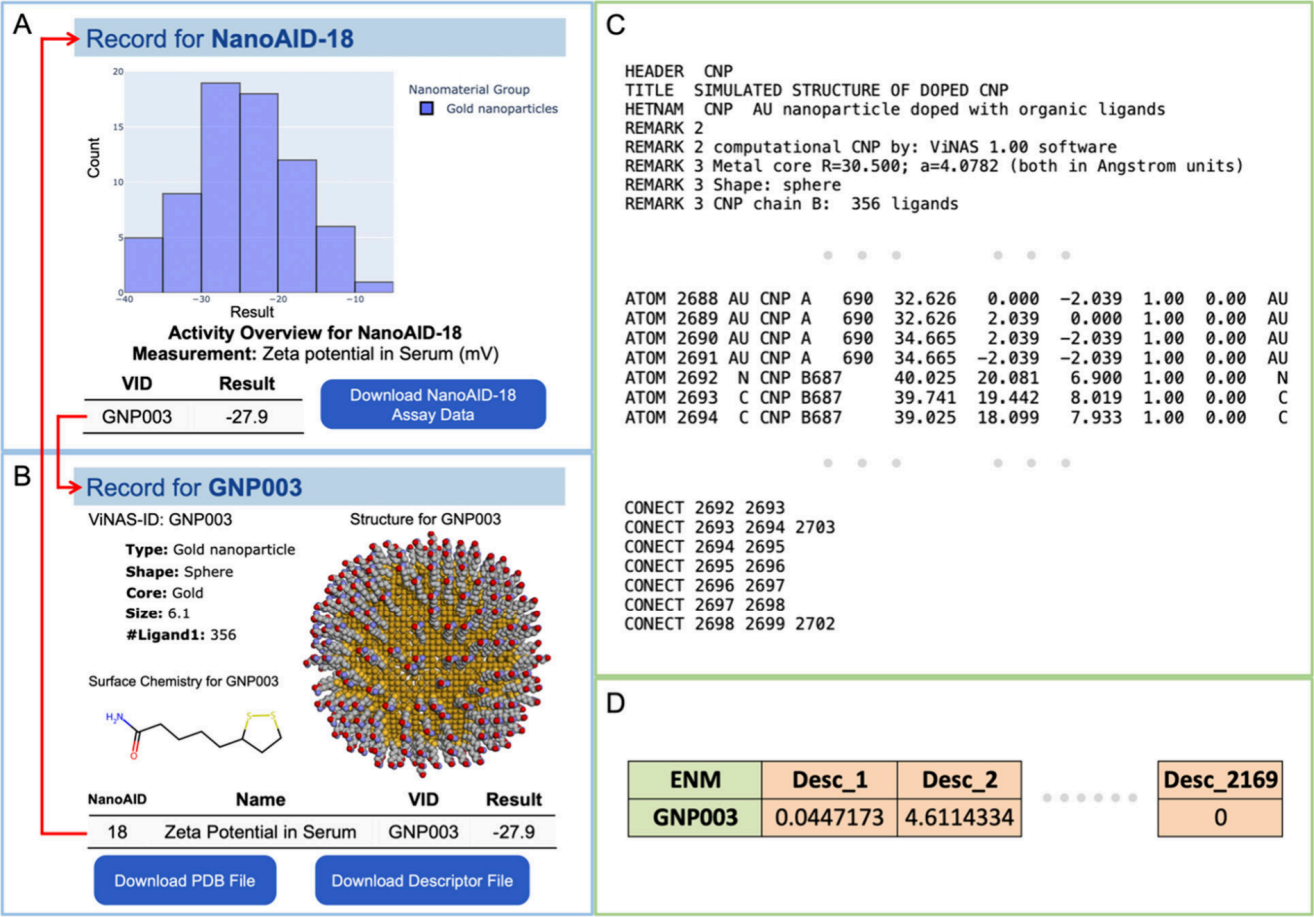
Integrating assay database and structure database for data profiling on ViNAS-Pro. (A) The assay record page provides an activity overview, associated NMs, and downloadable assay data for the corresponding assay. (B) The structure record page provides visualized nanostructure, nanostructure information, associated assays, and downloadable structure data as PDB format and nanodescriptor data as XLSX format. Two record pages are linked to each other by the interactive tables (red arrows in panels A and B). (C) An example PDB file is presented in three parts: basic information, atom types and coordinates, and connections between atoms. (D) An example nanodescriptor file contains 2169 nanodescriptor data calculated based on the annotated nanostructure.

Nanostructures are available in the structure database. For example, a record for GNP003, which belongs to the family of gold nanoparticles (GNPs), is shown in Figure 2B. GNP003’s record page provides its nanostructure figure rendering in van der Waals (VDW) format, along with basic structure information such as shape, size, core and ligand. Moreover, it includes an interactive table containing all the assay testing results associated with GNP003. The annotated nanostructure as a PDB file and nanodescriptor data as a XLSX file for GNP003 can also be downloaded from its record page. Example PDB file and nanodescriptor XLSX file are shown in Figure 2C,D. The PDB file, which stores annotated nanostructure information, consists of three sections: the first section presents fundamental details of the NM’s structure, such as form, shape, and size; the second section provides data of all the atoms, including atom type and coordinates; the last section details the bonds and connections between atoms. The nanodescriptor file consists of 2169 nanodescriptors, calculated based on the annotated nanostructure. Both the NM’s PDB file and nanodescriptor file are machine-readable for modeling purposes. Furthermore, users can navigate between related records by clicking on a specific assay in the interactive table on the structure record page to access the corresponding assay record page or a specific nanostructure material (NM) on the assay record page to access the nanostructure record page (Figure 2A,B). Connecting the assay database and nanostructure database in this way greatly improves the users’ abilities to find, analyze, and download data on ViNAS-Pro.

The Descriptor toolkit allows users to standardize nanodescriptor values using the descriptor preprocessing method and analyze the associated NM space using Principal Component Analysis (PCA). It provides two approaches for nanodescriptor analysis. The Descriptor List module allows users to analyze the nanodescriptors of target NMs on ViNAS-Pro. Users can selectively add the nanodescriptors of interest to the Descriptor List interface from the nanostructure record page. For example, the record page for a silver nanoparticle (AgNP), AgNP001, provides an interactive function to add its nanodescriptors to the Descriptor List page, as shown in Figure 3A. Subsequently, users can generate a customized descriptor list for specific NMs and submit it for further analysis, following the applications of preprocessing functions such as StandardScaler or MinMaxScaler (Figure 3A). The Descriptor Upload module allows users to upload their nanodescriptor data for analysis. For example, users can prepare their own nanodescriptor dataset for NMs in XLSX format, as shown in Figure 3B. They can then submit the nanodescriptor set for analysis (Figure 3B). The descriptor analysis results are shown on the descriptor analysis page (Figure 3C). Both two-dimensional (2D) and three-dimensional (3D) chemical spaces of NMs are shown by applying PCA to reduce the dimensionality of the nanodescriptors. Each dot represents a NM and provides the NM’s coordinates in the chemical space. The standardized nanodescriptor dataset, along with the 2D and 3D chemical space charts, can be downloaded on the descriptor analysis page. Compared with PubViNAS, the newly developed Descriptor Toolkit of ViNAS-Pro provides users new functions for data visualization and preprocessing, which can examine the structural diversity of the training data in the ML modeling procedure.

**Figure 3:**
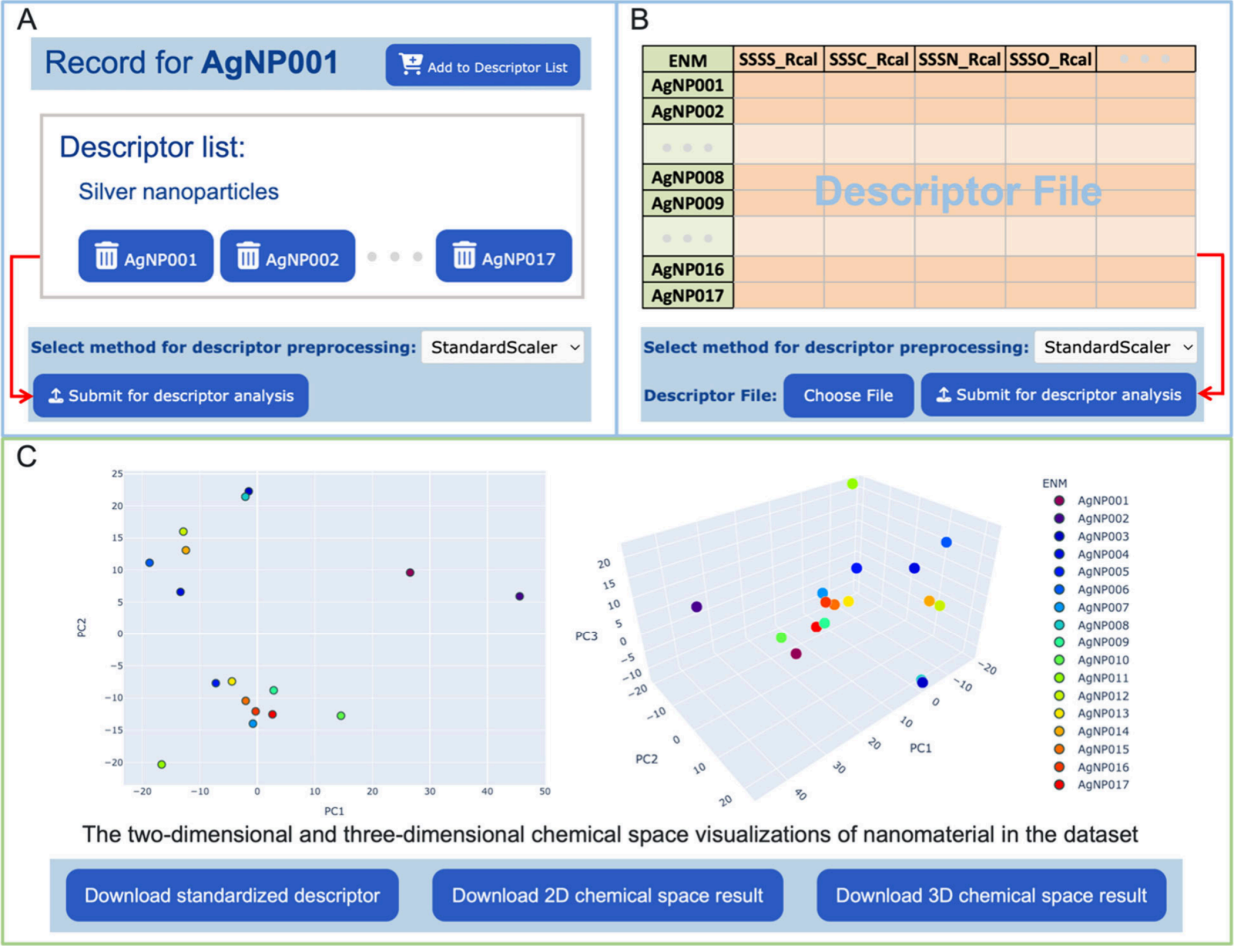
Descriptor preprocessing and chemical space visualization of NMs through Descriptor toolkit. (A) The Descriptor List approach and (B) the Descriptor Upload approach allow users to analyze NMs based on their nanodescriptors. (C) The descriptor analysis page provides analysis results, including the chemical space visualization charts, downloadable chemical space results, and a downloadable standardized nanodescriptor dataset.

The NanoPredictor module in the Model toolkit allows users to predict the properties, bioactivities, and toxicities of new NMs. It maintains a series of pre-developed ML models for different prediction tasks. For example, the NanoPredictor interface of the partial least-squares regression (PLSR) model developed for NMs with assay 19 (NanoAID-19) and assay 20 (NanoAID-20) data is shown in Figure S2A. Compared to PubViNAS, this page provides new information about the model descriptions, model-related literature, and an interactive scatter plot chart displaying the correlations between experimental and predicted values of the NMs used in the modeling. The interface allows for downloading the model file in pickle format, as well as the modeling datasets, including the nanodescriptor data and the assay data in XLSX format. Besides descriptions and downloadable data on the NanoPredictor interface, an overview of the modeling set for each NanoPredictor is shown in Table S3. An applicability domain is defined for each predictor, detailing the material type and size range of the dataset used for its development. This information assists users in selecting the appropriate predictor for NM prediction. Typically, new NMs with the same material type as those in the modeling set are considered to fall within the applicability domain. Users can locally prepare a nanodescriptor dataset of new NMs in XLSX format and submit it for prediction (Figure S2B). The prediction interface will employ the pre-developed model for predictions that are downloadable. Moreover, a dropdown menu is added to the module for switching between NanoPredictor interfaces with different pre-developed models, making it easy for users to perform various endpoint prediction tasks (Figure S2A).

As the upgraded predictive tool of the earlier PubViNAS, the AutoNanoML module was added to the Model toolkit to allow users to develop ML models through ViNAS-Pro. Two ML algorithms, linear regression (LR) and PLSR, are available for modeling in this module. For example, the initial AutoNanoML interface for developing the PLSR model is shown in Figure 4A. The modeling process can be divided into three steps: (1) uploading the descriptor and endpoint datasets in XLSX format; (2) choosing a descriptor standardization method, either StandardScaler or MinMaxScaler; and (3) selecting a cross-validation method among 3-Fold, 5-Fold, 10-Fold, or Leave-One-Out to develop the optimal ML model. After submission for modeling, the AutoNanoML interface will update with two new sections for model analysis and prediction (Figure 4B,C). As shown in Figure 4B, users can visualize the modeling results through an interactive scatter plot chart that illustrates the correlations between experimental and predicted values of the NMs used for modeling. In addition, they can analyze the nanodescriptors by exploring an interactive pie chart that illustrates the contributions of the top ranked descriptors derived from the modeling outcomes. The interface displays the optimal number of components for developing the best PLSR model, which is obtained from the cross-validation procedure. The determination (*R*^2^) and root-mean-square error (RMSE) are two key metrics for users to assess the model performance. The user developed models are downloadable, including the model file in pickle format, the scatter plot chart data, and the descriptor contribution data (Figure 4C). The prediction interface also enables users to use their developed model for prediction purposes (Figure 4C). The LR interface is similar to the PLSR interface, allowing users to develop LR models (Figure S3). The AutoNanoML module provides dataset selection and standardization, modeling and validation, and deployment of the model for prediction with downloadable outputs.

**Figure 4:**
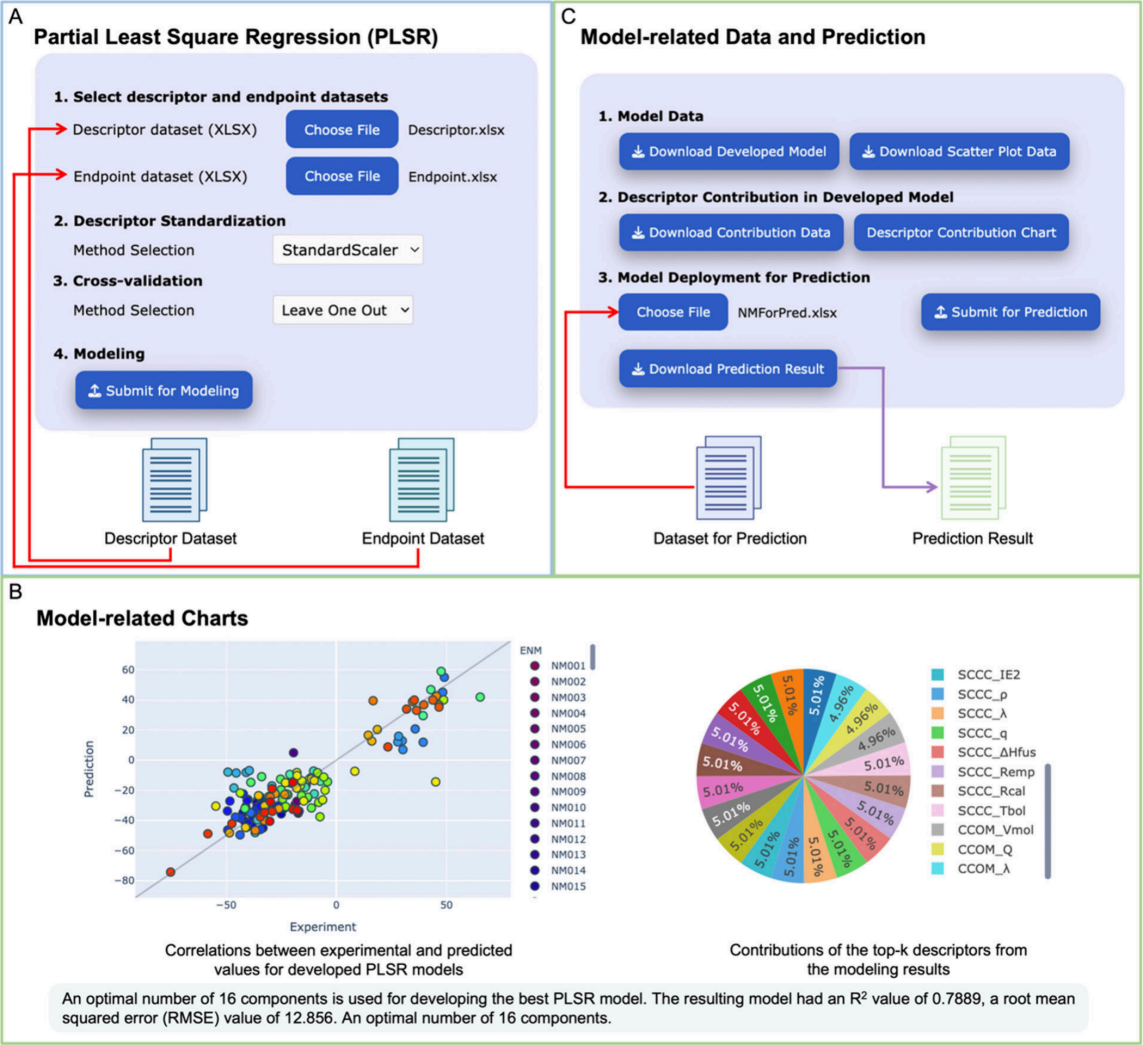
Developing the partial least-squares regression model for prediction through the AutoNanoML interface. (A) The initial interface allows users to upload a descriptor dataset and an endpoint dataset, as well as set up parameters for modeling. (B) After submitting the modeling task, the interface displays the model-related charts, such as a scatter plot demonstrating the correlation between experimental and predicted values of the NMs, and a pie chart illustrating the top-k descriptors’ contributions from the model results. Performance metrics, including the optimal number of components, *R*^2^, and RMSE, are displayed for model evaluation. (C) Model-related data, such as the model itself, the scatter plot chart data, and the descriptor contribution data, can be downloaded from the interface. Moreover, users can upload a nanodescriptor dataset in XLSX format for prediction by deploying the developed model.

To facilitate experimental research and reduce the time and cost associated with designing new NMs, a virtual NM library was constructed and integrated into ViNAS-Pro, consisting of diverse nanostructures along with predictions of their properties, bioactivities, and toxicities. This unique new function of ViNAS-Pro can greatly extend the current NM space, which was created through experimental synthesis, and provides users new NMs derived from developed machine learning models. The library currently consists of virtual NMs for three specific types: two-dimensional nanomaterials (2DNMs), platinum nanoparticles (PtNPs), and microplastics (MPs). In the construction of virtual 2DNMs, we rationalized essential structural parameters, such as size and surface modification, based on the eight synthesized 2DNMs to further develop 100 virtual 2DNM entities. Several representative newly designed virtual 2DNMs from the library are shown in Figure 5. These virtual 2DNMs were developed based on the structure features of eight synthesized 2DNMs, which defined the applicability domain of the relevant models, and exhibit diversity in size, shape, and surface modifications (e.g., carbon/oxygen ratio). They can be categorized as virtual graphene (v-G), virtual reduced graphene oxide (v-rGO), and virtual graphene oxide (v-GO), with their structure parameters (i.e., defined by the applicability domain) detailed in Table S4. Data Analysis of the virtual NMs is available through the Library Analysis module. The size distribution analysis reveals that the virtual 2DNMs fill the data gap, such as small size variety for the limited number of experimentally synthesized 2DNMs (Figure S4A). Based on the structures of virtual 2DNMs, geometrical nanodescriptors were calculated and then used to analyze the chemical space of these materials through PCA. The distribution of the library 2DNMs is visualized by using the top two or three principal components. In the 2D chemical space, these 2DNMs cluster into three groups: v-Gs, v-rGOs, and v-GOs (Figure S4B). In both the 2D and 3D chemical spaces, three groups of virtual 2DNMs exhibit intra-group structural diversity, effectively bridging the diversity gap resulting in experimentally synthesized 2DNMs as well (Figure S4B-E). Furthermore, we use the pre-developed ML models in the NanoPredictor module to predict the properties, bioactivities, and toxicities of virtual 2DNMs using their nanodescriptors as input. The predictions of the virtual 2DNMs are displayed in the interactive table of the Endpoint Profile interface, and users can batch download the prediction results (Figure S5A). Users can also choose specific virtual 2DNM in the table to access its detailed information. For example, the structure and basic information on v-rGO-010 are shown on its record page, where users can also download its structure data, descriptor data, and prediction results (Figure S5B). The construction of virtual PtNPs and virtual polystyrene (PS) MPs in the library is similar to that of the virtual 2DNMs (Figures S6–S9). The applicability domains used for their construction are shown in Table S5.

**Figure 5:**
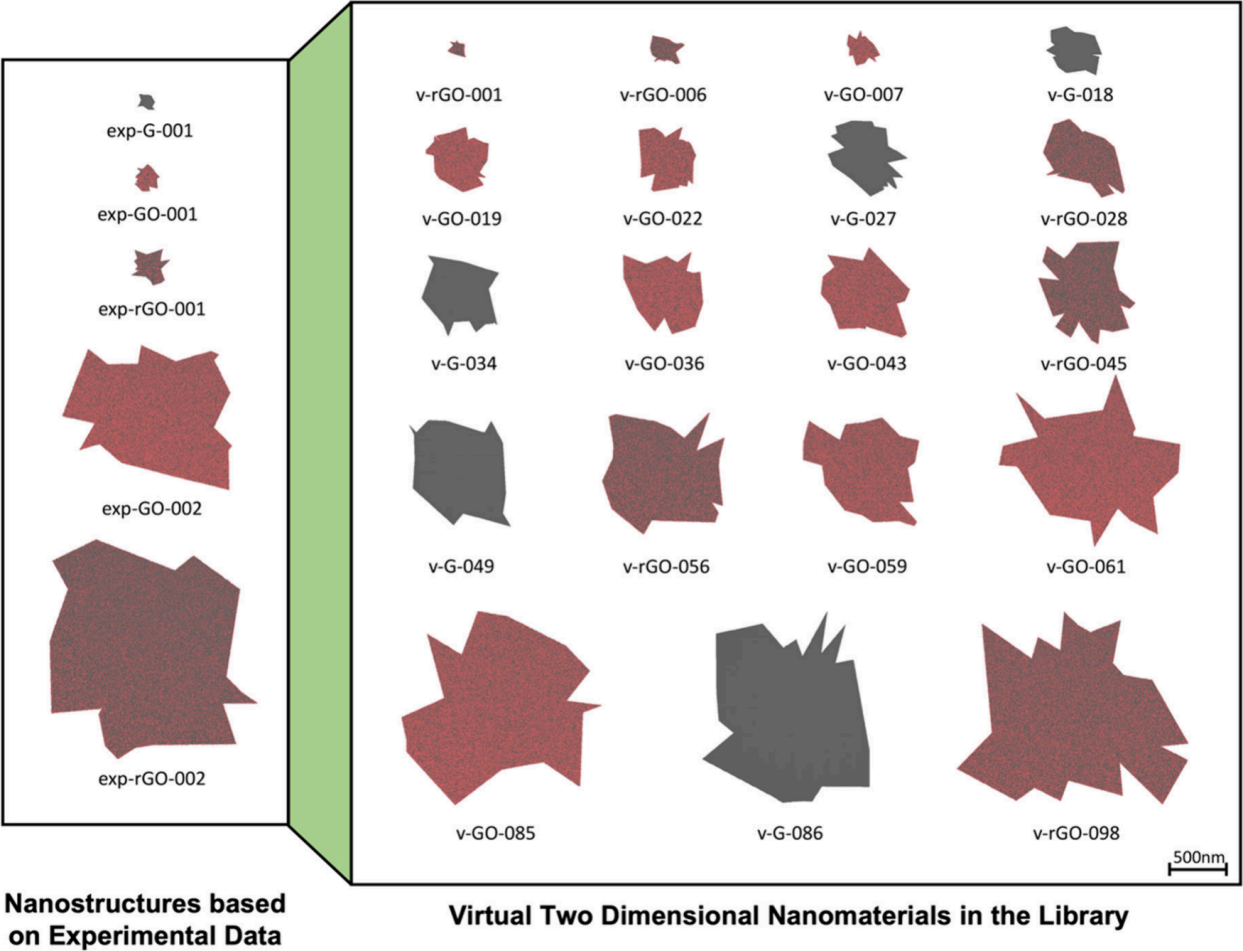
Visualization of representative virtual 2DNMs in the library. The virtual 2DNMs are constructed based on the structural features of experimentally synthesized 2DNMs, which exhibit diversity in size, shape, and surface modifications. The virtual nanostructures are rendered using the van der Waals drawing method in VMD, with carbon atoms in gray, oxygen atoms in red, and hydrogen atoms in blue.

In summary, we constructed a nanoinformatics platform that provides high-quality data, data analysis, ML modeling tools, endpoint predictions, and a large new virtual NM library, which can effectively facilitate the rational design of new NMs. ViNAS-Pro also provides services of data deposition and calculation (Figure S10). Furthermore, the Descriptor Upload module enables users to preprocess their own custom nanodescriptor data for specific NMs, such as experimentally measured and/or theoretically calculated nanodescriptors. The AutoNanoML module provides users with the flexibility to develop models using the custom nanodescriptor data and assay data of specific NMs. The components of ViNAS-Pro can be used in combination for various research tasks. A case study illustrating the design of NMs through ViNAS-Pro is shown in Figure S11. Registration for ViNAS-Pro is optional, but it will benefit users. Being registered users can enhance user support and services for modeling specific NMs as requested. In the future, we plan to integrate more NM data into ViNAS-Pro. More ML algorithms will be integrated into ViNAS-Pro for modeling purposes. Registered users can receive timely updates about critical ViNAS-Pro progress. The ViNAS-Pro platform will benefit from user feedback, and our future upgrade efforts aim to provide for the community an ultimate nanoinformatics platform similar to that for small molecules and proteins.

## Supporting Information Available

ViNAS-Pro architecture, the services of data deposit and calculation on ViNAS-Pro, case study using ViNAS-Pro for nanomaterial design, materials and methods, and supplemental figures and tables (PDF)
Structural parameters, nanostructure information, nanodescriptors, and predictions for nanomaterial used in the case study (ZIP)
