# Supplementary material for: An Online Nanoinformatics Platform Empowering Computational Modeling of Nanomaterials by Nanostructure Annotations and Machine Learning Toolkits
Source: Nano Lett. 2024 Aug 9;24(33):10228–36. doi: 10.1021/acs.nanolett.4c02568 (PMC11342361; doi:10.1021/acs.nanolett.4c02568)
Supplement: Supplementary file 1 — nl4c02568_si_001.pdf [file nl4c02568_si_001.pdf]

Supporting Information

An online nanoinformatics platform empowering computational modeling of nanomaterials by nanostructure annotations and machine learning toolkits

Tong Wang [a,b,c], *Daniel P. Russo* [c], *Philip Demokritou* [d,e], *Xuelian Jia* [a,b,c], *Heng Huang* [f], *Xinyu Yang* [c], *Hao Zhu* [a,b,c*]

[a] Tulane Center for Biomedical Informatics and Genomics, Tulane University, New Orleans, LA 70112, USA

[b] Division of Biomedical Informatics and Genomics, Deming Department of Medicine, Tulane University, New Orleans, LA 70112, USA

[c] Department of Chemistry and Biochemistry, Rowan University, Glassboro, NJ 08028, USA

[d] Center for Nanotechnology and Nanotoxicology, Department of Environmental Health, T.H. Chan School of Public Health, Harvard University, 655 Huntington Ave, Boston, MA 02115, USA

[e] Nanoscience and Advanced Materials Center, Environmental Occupational Health Sciences Institute, School of Public Health, Rutgers University, Piscataway, New Jersey 08854, USA

[f] Department of Computer Science, University of Maryland College Park, College Park, MD 20742, USA

[*] Corresponding author

E-mail address: hzhu10@tulane.edu

This PDF file includes:

ViNAS-Pro architecture, the services of data deposit and calculation on ViNAS-Pro, a case study using ViNAS-Pro for nanomaterial design

Materials and Methods (Online platform implementation; Experimental data collection and curation; Nanostructure annotation and nanodescriptor generation; Machine learning toolkits construction; Virtual nanomaterial library construction)

Figures S1 to S11, Tables S1 to S5, References

ViNAS-Pro architecture

The ViNAS-Pro platform comprises six core components (**Figure S1**). It maintains two machine readable databases: an assay database and a structure database. The assay database offers a profile with 5,069 data points covering properties, bioactivity, and toxicity for various NMs. The structure database contains structural information on 14 types of materials derived from experimental studies. Experimental data that describe the NMs' structures, including form, shape, size, and surface modifications, have been used to annotate nanostructures and shared through this database. Nanodescriptors have been calculated from these annotated nanostructures. The descriptor toolkit contains two modules: Descriptor List and Descriptor Upload, which can preprocess nanodescriptors for modeling purposes. The Model toolkit includes two modules: NanoPredictor and AutoNanoML. The NanoPredictor module maintains pre-developed ML models, enabling users to predict specific endpoints for new NMs. The AutoNanoML module provides an interface that allows users to develop their own ML models for various prediction purposes. The ViNAS-Pro library provides data analysis, data downloading, and endpoint predictions for virtual NMs through the Library Analysis and the

Endpoint Profile modules. ViNAS-Pro also provides services for data deposit, nanostructure construction, and nanodescriptor calculation through the Service component.

The services of data deposit and calculation on ViNAS-Pro

To facilitate data sharing within the nanoscience community, ViNAS-Pro provides a Data Deposit interface for users to deposit data into ViNAS-Pro databases (**Figure S10A**). Depositors can deposit nanostructure data in PDB format, as well as nanodescriptor data and assay data in CSV/XLSX format. After the in-house data cleaning and validation, the uploaded data will be integrated into the ViNAS-Pro databases. ViNAS-Pro provides a nanostructure construction and nanodescriptor calculation service for new NMs (**Figure S10B**). Users can request the service through the Calculation Service interface by providing basic information about the NM. When requesting nanodescriptor calculation, users are encouraged to provide corresponding nanostructures in PDB format. Afterwards, users will receive a calculation result by email, which can be used for modeling and other nanoinformatics tasks.

A case study using ViNAS-Pro for nanomaterial design

A case study that described the design of a new gold nanoparticle is shown in **Figure S11**. Firstly, the user needs to set up the structural parameters including material type, shape, size, core, ligand SMILES, and ligand density, for constructing a desired GNP (**Figure S11A**). Then this information will be submitted through the Calculation Service interface (https://vinas-toolbox.com/calculation_service). Then the user will receive a PDB file storing the nanostructure information and an excel file for nanodescriptors (**Figure S11B**). The user can use pre-developed models on the NanoPredictor interface to predict the properties and bioactivities of this new GNP by uploading the calculated nanodescriptors, such as ROS in A549 cells and

zeta potential in water from NanoAID-15 (https://vinas-toolbox.com/model_developed_aid15) and 16 (https://vinas-toolbox.com/model_developed_aid16) (**Figure S11C**). Moreover, the user can also develop and use their own models for predictions as introduced in the Document Tutorial on ViNAS-Pro (https://vinas-toolbox.com/tutorial). In the end, this new GNP can be experimentally synthesized with desired predicted properties/activities (**Figure S11D**). The related data for this case study are also available in the supporting information.

Materials and Methods

Online platform implementation

The backend of ViNAS-Pro relies on the Python-based Flask framework to manage server-side logic, handling user requests and generating operations based on requests. On the frontend, HTML and JavaScript were employed to create the user interface. A variety of open-source libraries, such as SQLite, 3Dmol, DataTables, and Plotly, were used for data visualization and manipulation during data analysis and modeling process. These open-source libraries were added to support necessary functions of ViNAS-Pro and can be updated easily when there are newer libraries available in the future.

Experimental data collection and curation

The ViNAS-Pro database was compiled with 328 unique NM records from in house studies and 422 unique NM records from external data. The external data were manually collected from literatures and most of these data have been used in our previous modeling studies [1-3]. To ensure the quality, the data have been incorporated into the database under the following conditions: (1) basic information about NMs, such as core shape and size, was provided in the original sources; (2) surface chemistry information was included, and the surface ligand structure

can be annotated in Simplified Molecular Input Line Entry System (SMILES) format; (3) property/bioactivity/toxicity data were available for each NM record. The details of data curation process were described in our previous studies [1, 2].

Nanostructure annotation and nanodescriptor generation

For the structure annotation of nanoparticles, the core atoms were initially assembled into a nano core based on the particle size and shape information. Subsequently, the associated surface ligands/atoms were randomly distributed on the core surface [1, 2]. All annotated nanostructures on ViNAS-Pro were saved in PDB format. Due to their large sizes, we employed a size scaling-down technique to construct microplastic structures, which facilitated the annotation and storage of their structural information in PDB files [2]. For example, we reduced the sizes of four microplastics (MP001 to MP004) in the dataset (NanoAID-27) by a factor of 70 to improve the efficiency of structural constructions. On the corresponding NM record page and assay page, we provided the relevant information for users as well. In our previous studies, we developed novel geometrical nanodescriptors by employing Delaunay tessellation and atomic properties [2, 4]. Every four nearest atoms that can form a tetrahedron were identified as nanodescriptor from nanostructures. These nanodescriptors can quantify nanostructures by simulating NMs' surface chemistry for modeling purposes. Nanodescriptors for each NM records on ViNAS-Pro were calculated using in-house scripts (coded in C++/Java 1.8.0_301) and saved in XLSX format.

Machine learning toolkits construction

Descriptor and Model toolkits were developed by Python and various Python libraries such as scikit-learn. The Descriptor toolkit incorporated the PCA method to transform high-

dimensional data into lower-dimensional representations, enabling users to analyze and visualize the chemical space of NMs. The Model toolkit's NanoPredictor module provides in-house ML models for predictions, and the AutoNanoML module implements LR and PLSR algorithms for ML modeling. LR is a classic algorithm for developing regression models that predict various endpoints of NMs, such as cellular uptake, viability, apoptosis, and oxidative stress [5, 6]. PLSR, combining PCA and multiple linear regression, reduces descriptor dimensions and constructs components to account for dataset variance, helping avoid multicollinearity and overfitting. It is suitable for modeling small training sets with large descriptor sets. The cross-validation procedure was implemented to find the optimal parameters for modeling. The coefficient of R^2 and RMSE were used as key metrics to evaluate the resulted model as described in our previous study [2].

Virtual nanomaterial library construction

To construct the virtual NM library, we created an NM dataset containing basic structural information by rationalizing structural parameters. The range of these parameter values were based on the same parameters obtained from experimental details of references. The virtual nanostructures that have never been synthesized were constructed using in-house scripts (coded in Python 3.8), which took the basic structure information from the library dataset as input parameters. The annotated nanostructures in the library were saved in PDB formats. Geometrical nanodescriptors were calculated from generated nanostructures using in-house scripts (C++/Java 1.8.0_301), and these descriptors were used with pre-developed ML models to predict the properties, bioactivities, and toxicities of new NMs in the library.

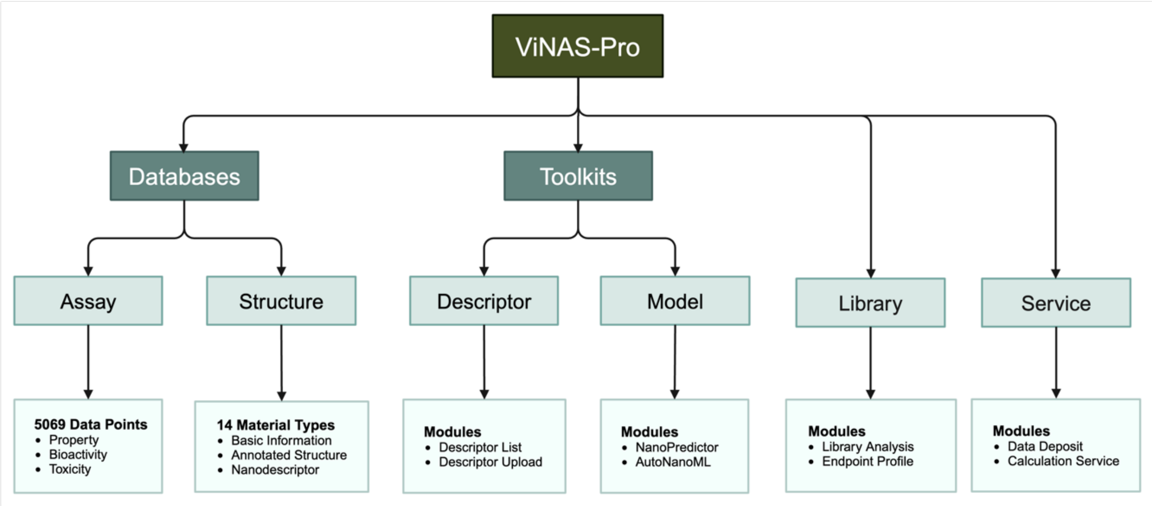

Figure S1. Schematic overview of the ViNAS-Pro platform architecture. ViNAS-Pro consists of six components, including Assay database, Structure database, Descriptor toolkit, Model toolkit, Library, and Service.

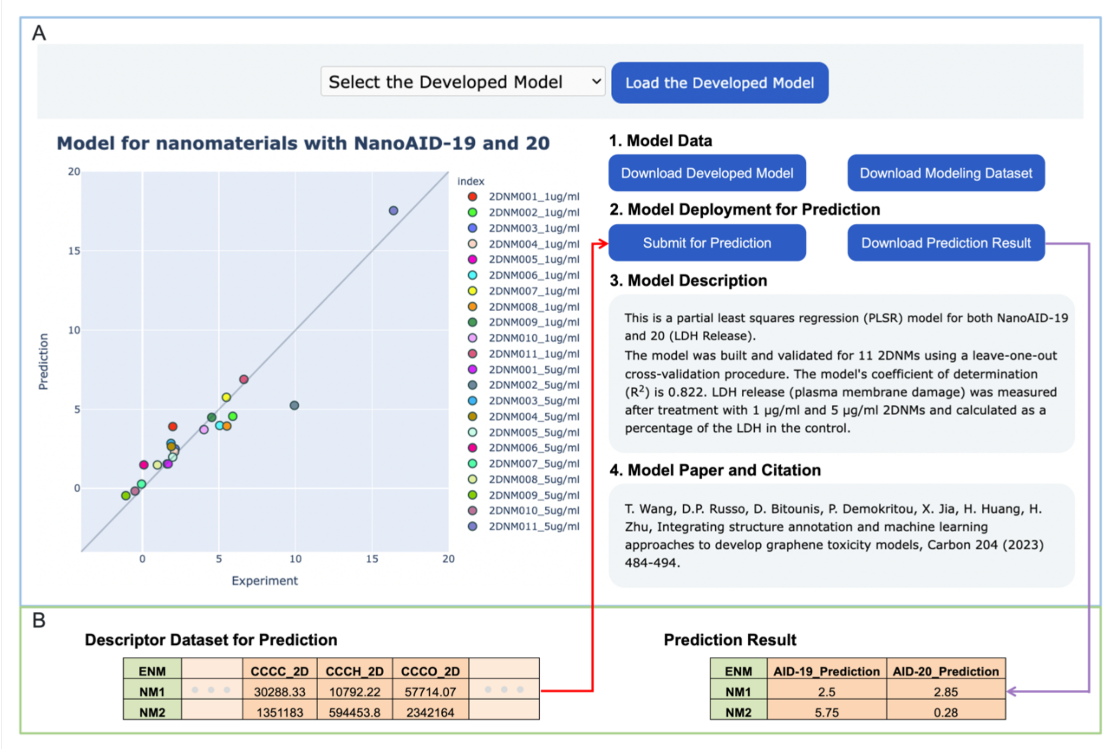

Figure S2. Performing prediction by the pre-developed models through the NanoPredictor interface. (**A**) an example of NanoPredictor interface provides a model description, model-

related literature, and downloadable model data. Users can switch between different NanoPredictor interfaces by selecting and loading a model from the dropdown menu. (**B**) The NanoPredictor interface allows users to submit a descriptor dataset for new NMs (red arrow) and provides predictions by employing the pre-developed model on the interface (purple arrow).

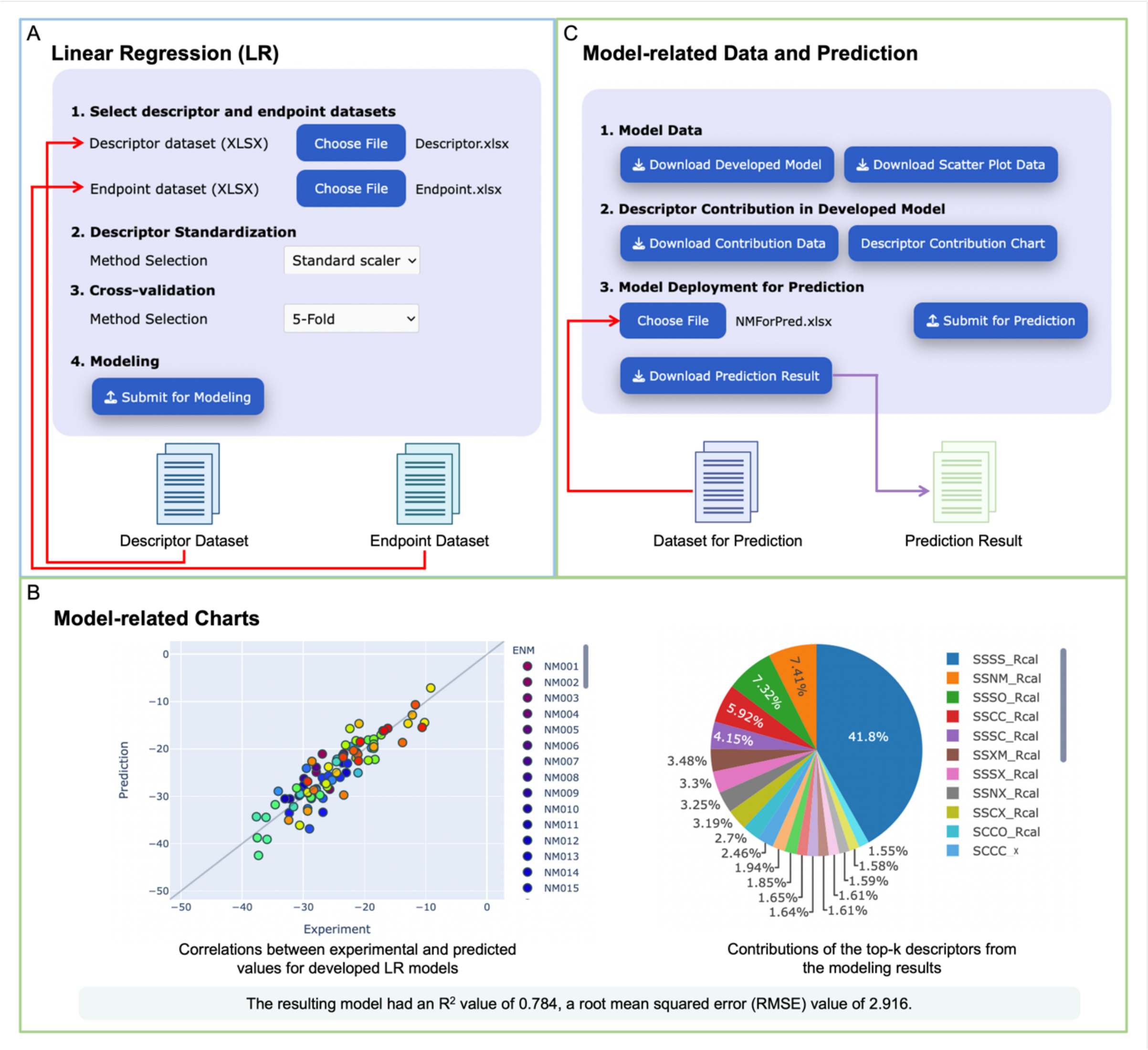

Figure S3. Developing the linear regression model for prediction through the AutoNanoML interface. (**A**) The initial interface allows users to upload a descriptor dataset and an endpoint dataset, as well as set up parameters for modeling. (**B**) After submitting the modeling task, the

interface displays the model-related charts, such as a scatter plot demonstrating the correlation between experimental and predicted values of the NMs, and a pie chart illustrating the top-k descriptors' contributions from the model results. Performance metrics, including R^2 and RMSE, are displayed for model evaluation. (**C**) Model-related data, such as the model itself, the scatter plot chart data, and the descriptor contribution data, can be downloaded from the interface. Moreover, users can upload a nanodescriptor dataset in XLSX format for prediction by deploying the developed model.

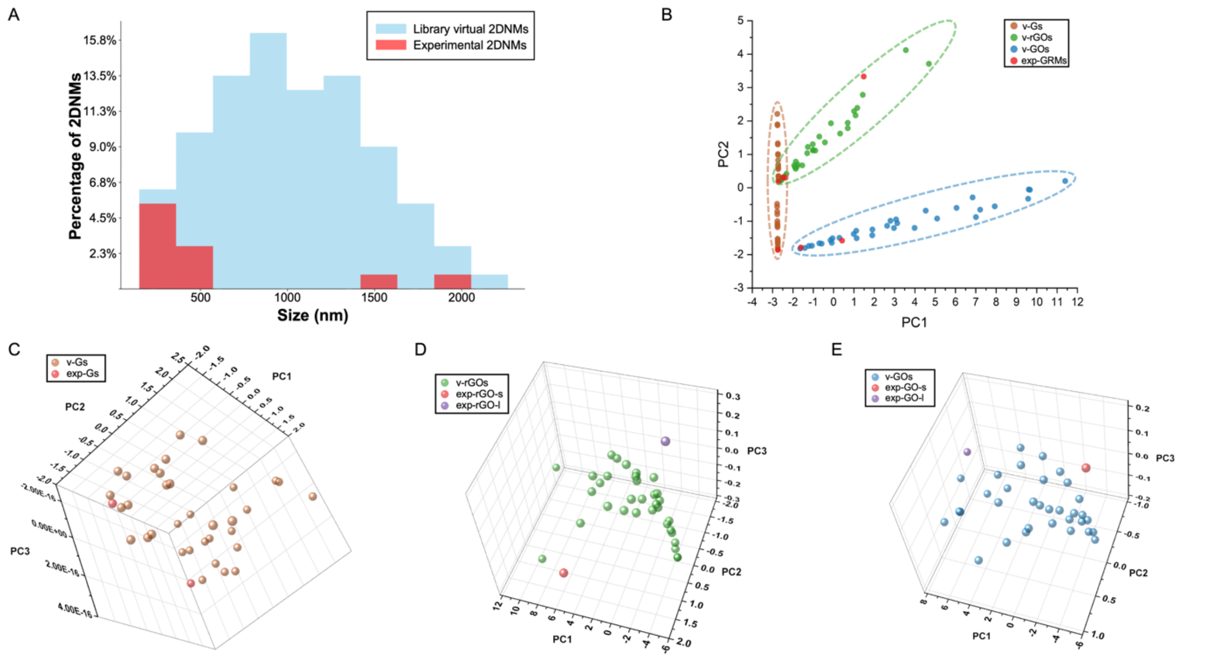

Figure S4. Exploratory data analysis of virtual 2DNMs in the library. (**A**) The histogram shows the size distributions of both experimentally synthesized 2DNMs and virtual 2DNMs in the library. (**B-D**) In both the 2D and 3D chemical spaces, the virtual 2DNMs show diversity, effectively bridging the structural gap caused by the limited number of experimental 2DNMs. Abbreviations: v-Gs (virtual graphenes), v-rGOs (virtual reduced graphene oxide), v-GOs (virtual graphene oxide), exp-GRMs (experimental graphene-related materials), exp-Gs

(experimental graphenes), exp-rGO-s (experimental reduced graphene oxide in small size), exp-rGO-l (experimental reduced graphene oxide in large size), exp-GO-s (experimental graphene oxide in small size), exp-GO-l (experimental graphene oxide in large size).

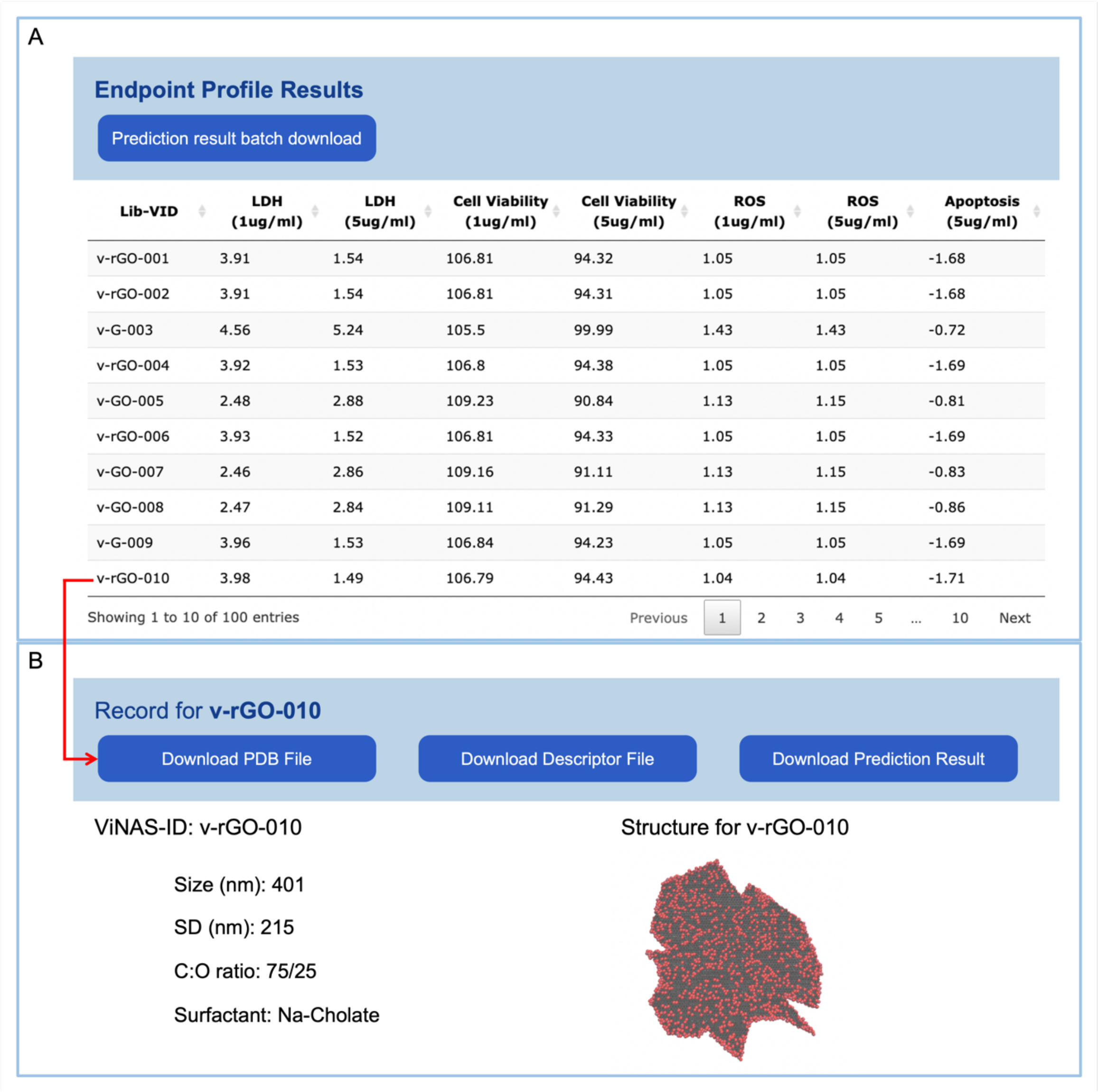

Figure S5. Endpoint Profiling of virtual 2DNMs in the library. (**A**) Users can access the predictions of properties/bioactivities/toxicities for virtual 2DNMs through the Endpoint Profile interface, with options to download these results in batches. (**B**) By clicking on a specific virtual 2DNM in the interactive table, users will be directed to its detailed record page (red arrow). This

page displays the 2DNM’s structural information and provides downloadable access to its structure data, descriptor data, and prediction results.

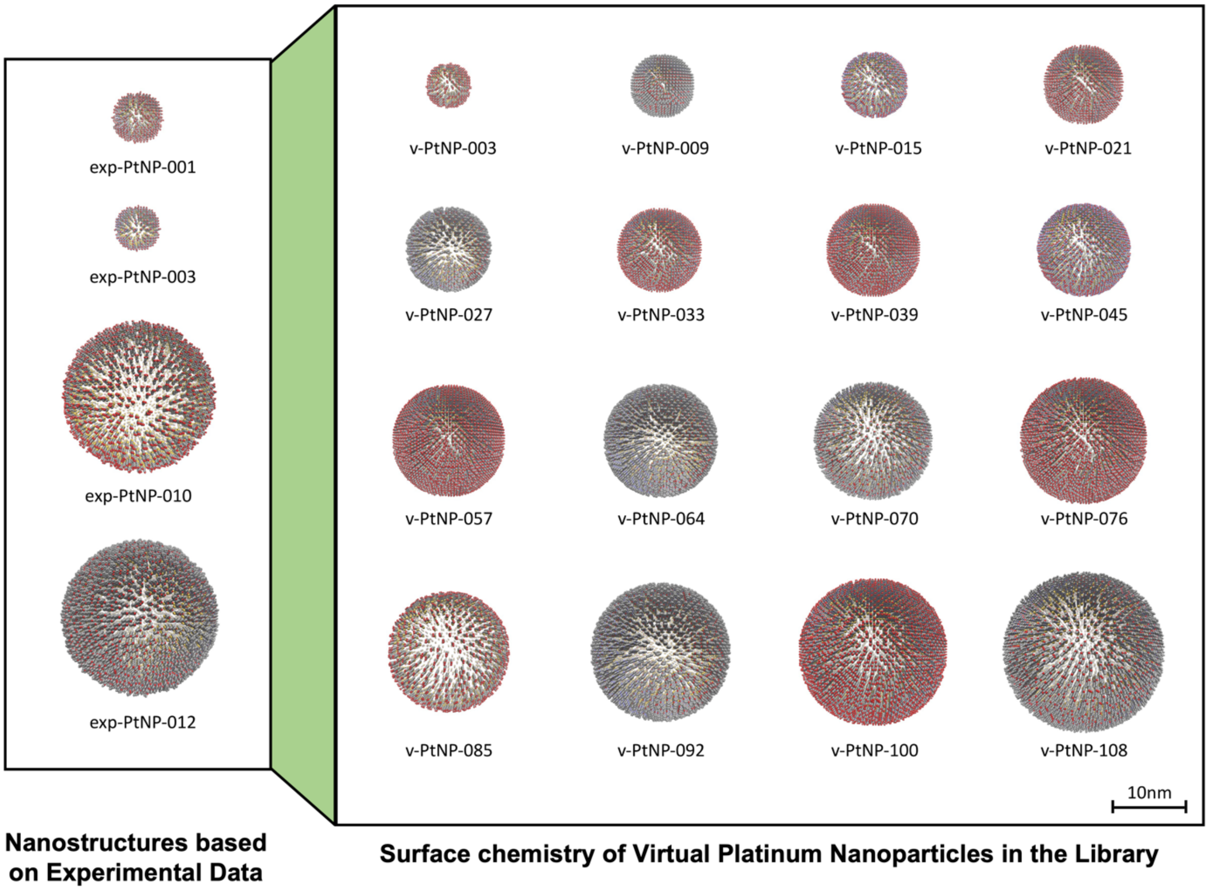

Figure S6. Visualization of representative virtual PtNPs in the library. The virtual PtNPs are constructed based on the structural features of experimentally synthesized PtNPs, which exhibit diversity in size, types of surface ligands, and the number of surface ligands. The virtual nanostructures are rendered using the VDW drawing method in VMD, with carbon atoms in grey, oxygen atoms in red, nitrogen atoms in blue, and sulfur atoms in yellow.

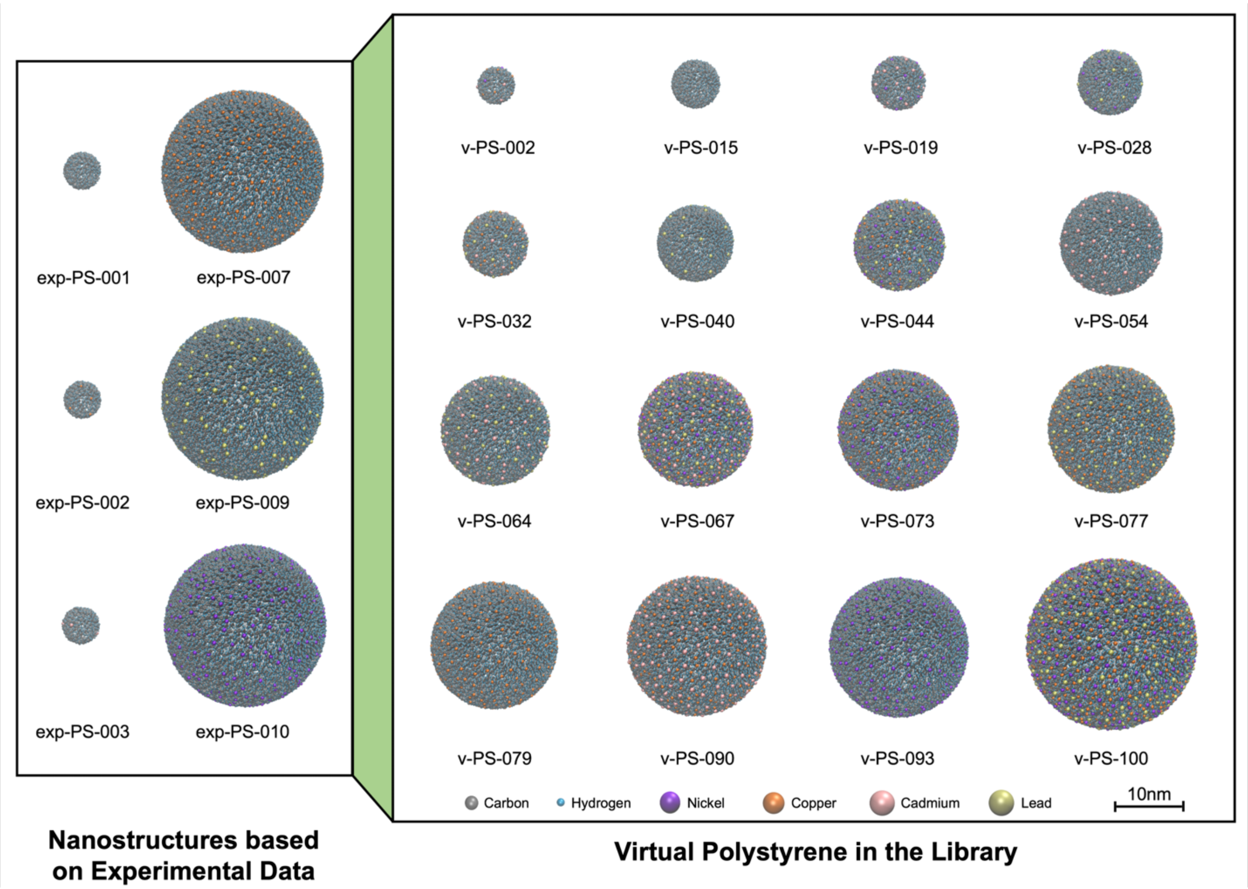

Figure S7. Visualization of representative virtual PS in the library. The virtual PS are constructed based on the structural features of experimentally synthesized PS, which exhibit diversity in size and surface modification. For surface modification, the virtual PS surface can adsorb one or more of four types of heavy metals, including nickel, copper, cadmium, and lead. The number of heavy metals on the surface of virtual PS is also different. The virtual nanostructures are rendered using the VDW drawing method in VMD.

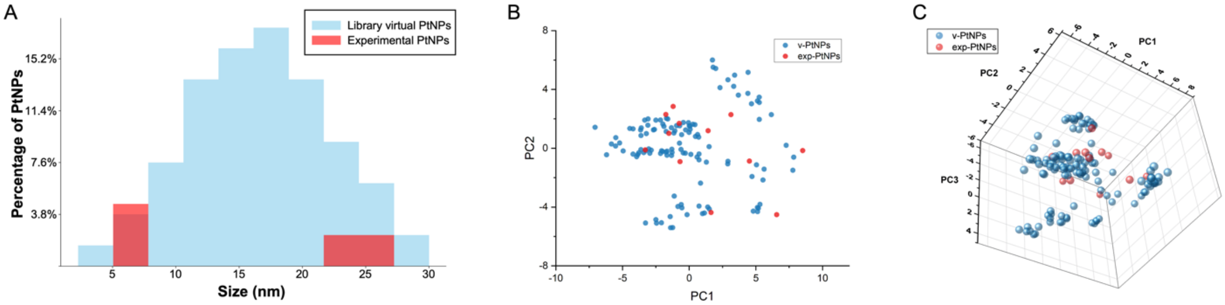

Figure S8. Exploratory data analysis of virtual PtNPs in the library. (**A**) The histogram shows the size distributions of both experimentally synthesized PtNPs and virtual PtNPs in the library. (**B, C**) In both the 2D and 3D chemical spaces, the virtual PtNPs show diversity, effectively bridging the structural gap caused by the limited number of experimental PtNPs. Abbreviations: v-PtNPs (virtual PtNPs), exp-PtNPs (experimental PtNPs).

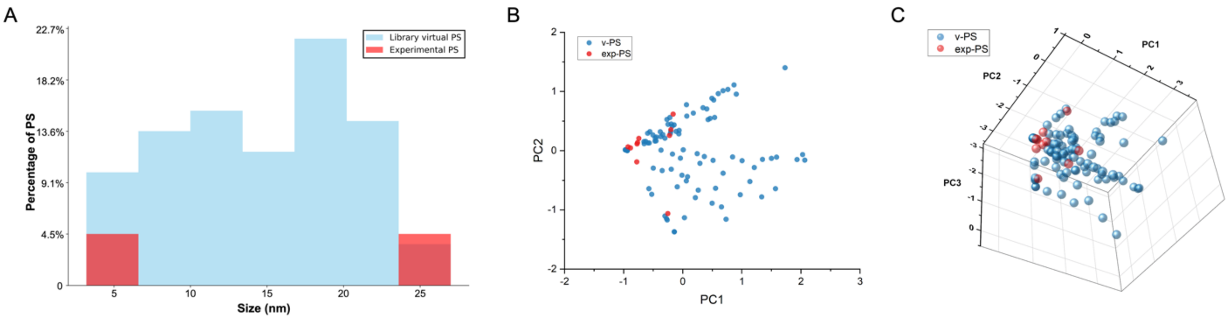

Figure S9. Exploratory data analysis of virtual PS in the library. (**A**) The histogram shows the size distributions of both experimentally synthesized PS and virtual PS in the library. (**B, C**) In both the 2D and 3D chemical spaces, the virtual PS show diversity, effectively bridging the structural gap caused by the limited number of experimental PS. Abbreviations: v-PS (virtual PS), exp-PS (experimental PS).

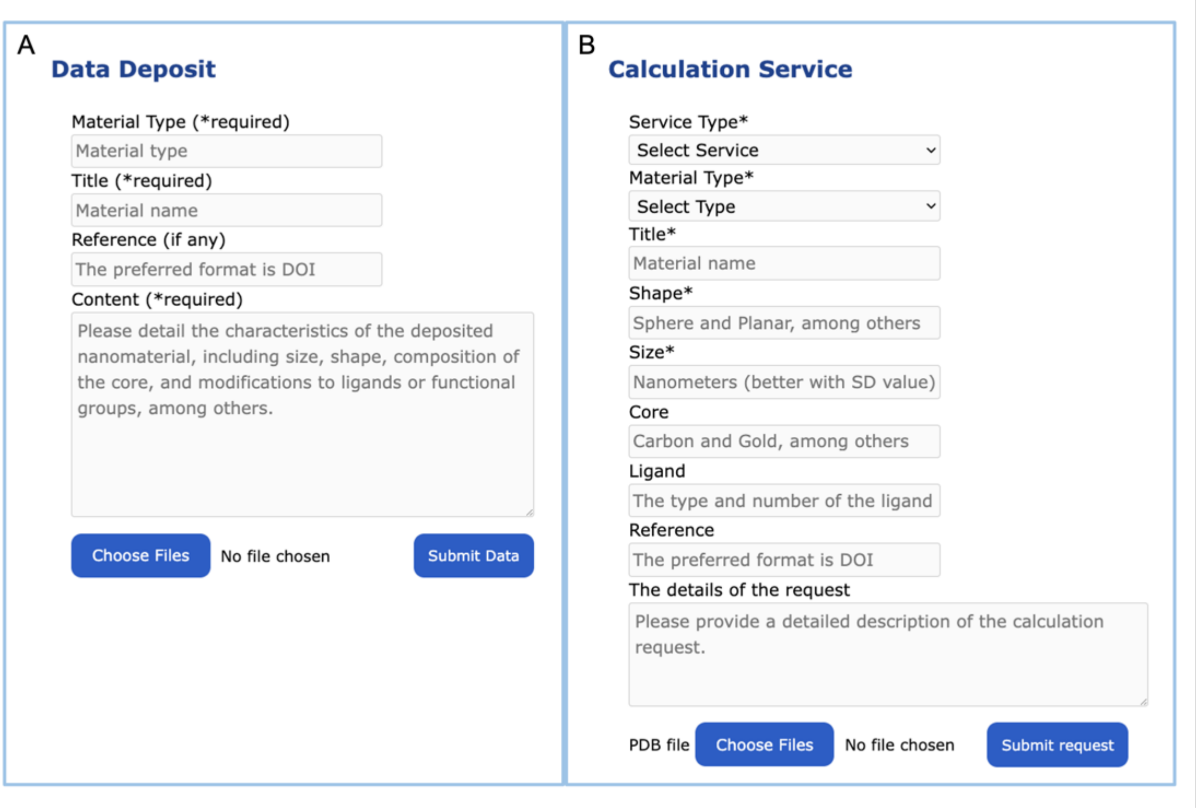

Figure S10. Overview of services provided by ViNAS-Pro. (**A**) The Data Deposit interface and (**B**) the Calculation Service interface allow users to deposit data and request the construction of nanostructures and the calculation of nanodescriptors for new NMs.

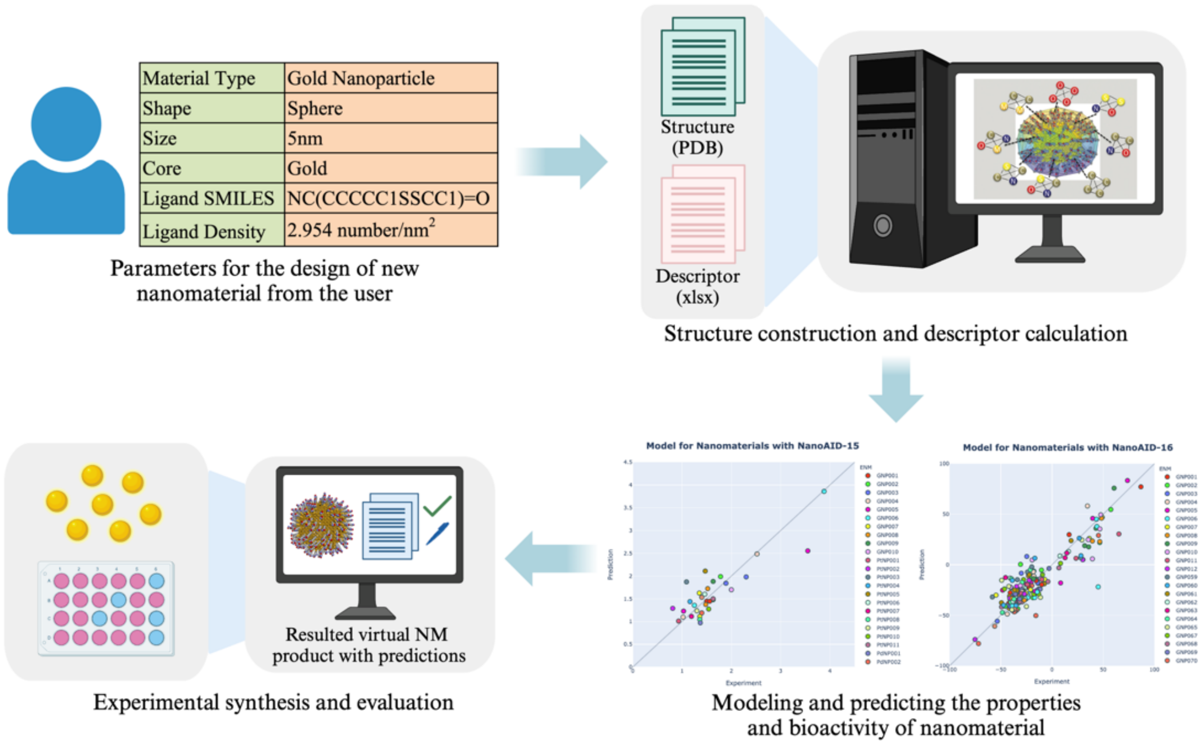

Figure S11. A case study on designing new nanomaterials using ViNAS-Pro. (A) Users request the calculation of new nanomaterials by uploading corresponding parameters on the Calculation Service interface. (B) Calculation results, including structural and nanodescriptor data for the new nanomaterial, are sent to users by email. (C) Based on the calculated nanodescriptors, users can predict the properties and bioactivity of new nanomaterials by employing pre-developed models on the NanoPredictor interface or use their own models. (D) The new nanomaterials can be experimentally synthesized and tested based on the corresponding virtual nanomaterial generated from ViNAS-Pro.

Table S1. Comparison of representative online nanomaterial databases

Database feature	caNanoLab [7]	eNanoMapper [8]	NanoInformaTIX [9]	NaKnowBase [10]	NIKC [9]	PubVINAS [1]	ViNAS-Pro
Publicly available	Limited	Yes	Limited	Yes	Yes	Yes	Yes
Property/activity/toxicity data	Yes	Yes	Yes	Yes	Yes	Yes	Yes
Nanostructure annotation	No	No	No	No	No	Yes	Yes
Online data analysis	No	No	Yes	No	Yes	No	Yes
Online predictors for predictions	No	No	Yes	No	Yes	No	Yes
Online model developing tools	No	No	No	No	No	No	Yes
New virtual nanomaterial library	No	No	No	No	No	No	Yes

Table S2. Overview of ViNAS-Pro assays

NanoAID	Assay	Organism	Cell line/Tissue	Endpoint category
1	Non-specific binding with AChE enzyme	Mouse	Blood	Human health
2	Specific binding with AChE enzyme	Mouse	Blood	Human health
3	Autophagy induction	Human	THP-1 (monocyte)	Human health
4	Cellular association	Human	A549 cells	Human health
5	Cellular uptake I	Human	A549 cells	Human health
6	Cellular uptake II	Human	A549 cells	Human health
7	Cellular uptake III	Human	HEK293 cells	Human health
8	Cell viability I	Human	THP-1 (monocyte)	Human health
9	logP	Human	A549 and 16HBE cells	Human health
10	Metabolic activity of CYP3A4	Human	Liver microsomes	Human health
11	PFOS adsorption	N/A	N/A	Environmental concerns
12	Protein adsorption	Bovine	Serum	Human health
13	ROS I	Human	HEK293 cells	Human health
14	ROS II	Human	A549 cells	Human health
15	ROS III	Human	A549 cells	Human health
16	Zeta potential I	N/A	N/A	Property
17	Zeta potential II	N/A	N/A	Property
18	Zeta potential III	N/A	N/A	Property
19	LDH release I	Human	Caco-2, HT29-MTX, and Raji B cells	Human health
20	LDH release II	Human	Caco-2, HT29-MTX, and Raji B cells	Human health
21	Cell viability II	Human	Caco-2, HT29-MTX, and Raji B cells	Human health
22	Cell viability III	Human	Caco-2, HT29-MTX, and Raji B cells	Human health
23	ROS IV	Human	Caco-2, HT29-MTX, and Raji B cells	Human health
24	ROS V	Human	Caco-2, HT29-MTX, and Raji B cells	Human health
25	Apoptosis	Human	Caco-2, HT29-MTX, and Raji B cells	Human health
26	Immobilization rate (EC50)	Daphnia magna	Aquatic Organisms	Environmental concerns
27	Zeta potential IV	N/A	N/A	Property

Note: PFOS: Perfluorooctanesulfonic acid; ROS: Reactive oxygen species; LDH: Lactate dehydrogenase; EC50: Half maximal effective concentration; N/A: Not applicable.

Table S3. Overview of modeling set for NanoPredictors on ViNAS-Pro

Model	Number of NMs	NM type (size range)
Model for NanoAID-9	147	GNP (3.63-27.78nm), PtNP (5.4-25.3nm), PdNP (5.4-26.76nm)
Model for NanoAID-12	36	GNP (6.1-27.78nm), PtNP (5.4-25.3nm), PdNP (5.4-26.76nm)
Model for NanoAID-15	36	GNP (6.1-27.78nm), PtNP (5.4-25.3nm), PdNP (5.4-26.76nm)
Model for NanoAID-16	213	GNP (1.5-33nm), AgNP (9.9-15nm), PtNP (5.4-25.3nm), PdNP (5.4-26.76nm), MONP (8-19nm), QDNP (2.4-10nm), Dendrimer (4.5-6.7nm)
Model for NanoAID-19 and 20	11	Graphene-related 2DNM (184-2015nm), Inorganic 2DNM (149-428nm)
Model for NanoAID-21 and 22	11	Graphene-related 2DNM (184-2015nm), Inorganic 2DNM (149-428nm)
Model for NanoAID-23 and 24	11	Graphene-related 2DNM (184-2015nm), Inorganic 2DNM (149-428nm)
Model for NanoAID-25	9	Graphene-related 2DNM (184-2015nm), Inorganic 2DNM (149-428nm)
Model for NanoAID-26	10	MP (5-25nm)

Note: GNP: gold nanoparticle; AgNP: silver nanoparticle; PtNP: platinum nanoparticle; PdNP: palladium nanoparticle; MONP: metal oxides nanoparticle; QDNP: quantum dot; 2DNM: two-dimensional nanomaterial; MP: microplastic.

Table S4. Applicability domain for the construction of virtual 2DNMs

Virtual 2DNMs	Size (nm)	Carbon/Oxygen ratio	Surfactant
v-Gs	37-555	N/A	PF108/Na-Cholate
v-rGOs	76-521	1.56-1.77	Na-Cholate
v-GOs	79-466	2.57-3.55	Water

Note: The sizes of virtual 2DNMs are determined by their lateral size. The surfactants used for v-Gs can be either PF108 or Na-Cholate. N/A: Not applicable.

Table S5. Applicability domain for the construction of virtual PtNPs and MPs

Virtual Nanoparticles	Size (nm)	Ligand type (SMILES)	Ligand density (number/nm^2)
v-PtNPs	2.29-30.01	NC(CCCCC1CCSS1)=O	0-3.370
		O=C(NC1=CC=CC=C1)CCCCC2CCSS2	0-3.703
		O=C(NCCCC)CCCCC1CCSS1	0-6.037
		O=C(NCCOCCO)CCCCC1CCSS1	0-6.575
		OC(CCCCC1CCSS1)=O	0-3.895
		OCCNC(CCCCC1CCSS1)=O	0-5.892
v-PS	5-25	[Cu]	0-0.500
		[Cd]	0-0.243
		[Pb]	0-0.188
		[Ni]	0-0.295

Note: The sizes of virtual PtNPs (v-PtNPs) and MPs (v-PS) are determined by their diameters. For v-PtNPs, each particle displays only one type of ligand on its surface. For v-PS, each particle can display either a mixture of ligands or no ligands on its surface.

References

(1) Yan, X.; Sedykh, A.; Wang, W.; Yan, B.; Zhu, H. Construction of a web-based nanomaterial database by big data curation and modeling friendly nanostructure annotations. *Nature communications* **2020,** *11* (1), 2519.

(2) Wang, T.; Russo, D. P.; Bitounis, D.; Demokritou, P.; Jia, X.; Huang, H.; Zhu, H. Integrating structure annotation and machine learning approaches to develop graphene toxicity models. *Carbon* **2023,** *204*, 484-494.
(3) Russo, D. P.; Yan, X.; Shende, S.; Huang, H.; Yan, B.; Zhu, H. Virtual molecular projections and convolutional neural networks for the end-to-end modeling of nanoparticle activities and properties. *Analytical Chemistry* **2020,** *92* (20), 13971-13979.
(4) Yan, X.; Sedykh, A.; Wang, W.; Zhao, X.; Yan, B.; Zhu, H. In silico profiling nanoparticles: predictive nanomodeling using universal nanodescriptors and various machine learning approaches. *Nanoscale* **2019,** *11* (17), 8352-8362.
(5) Furxhi, I.; Murphy, F.; Mullins, M.; Arvanitis, A.; Poland, C. A. Practices and trends of machine learning application in nanotoxicology. *Nanomaterials* **2020,** *10* (1), 116.
(6) Li, J.; Wang, C.; Yue, L.; Chen, F.; Cao, X.; Wang, Z. Nano-QSAR modeling for predicting the cytotoxicity of metallic and metal oxide nanoparticles: A review. *Ecotoxicology and Environmental Safety* **2022,** *243*, 113955.
(7) Gaheen, S.; Hinkal, G. W.; Morris, S. A.; Lijowski, M.; Heiskanen, M.; Klemm, J. D. caNanoLab: data sharing to expedite the use of nanotechnology in biomedicine. *Computational science & discovery* **2013,** *6* (1), 014010.
(8) Jeliazkova, N.; Chomenidis, C.; Doganis, P.; Fadeel, B.; Grafström, R.; Hardy, B.; Hastings, J.; Hegi, M.; Jeliazkov, V.; Kochev, N. The eNanoMapper database for nanomaterial safety information. *Beilstein journal of nanotechnology* **2015,** *6* (1), 1609-1634.
(9) Amos, J. D.; Tian, Y.; Zhang, Z.; Lowry, G. V.; Wiesner, M. R.; Hendren, C. O. The NanoInformatics Knowledge Commons: Capturing spatial and temporal nanomaterial transformations in diverse systems. *NanoImpact* **2021,** *23*, 100331.
(10) Mortensen, H. M.; Beach, B.; Slaughter, W.; Senn, J.; Williams, A.; Boyes, W. Translating nanoEHS data using EPA NaKnowBase and the resource description framework. *F1000Research* **2024,** *13*.
